# Evaluation of Different Processes Impact on Flavor of Camellia Seed Oil Using HS-SPME-GC/MS

**DOI:** 10.3390/molecules28103979

**Published:** 2023-05-09

**Authors:** Ziming Li, Xiangyu Zhou, Hongai Li, Wenhua Zhou, Yuheng Tan, Yuxin Zhang, Jiarong She, Jun Lu, Ninghua Yu

**Affiliations:** 1 Hunan Key Laboratory of Forestry Edible Sources Safety and Processing, Central South University of Forestry and Technology, Changsha 410004, China; 20200100085@csuft.edu.cn (Z.L.); zhouwenhua@126.com (W.Z.);; 2 Hunan Key Laboratory of Processed Food for Special Medical Purpose, Changsha 410004, China; 3 Faculty of Medical Science, Division of Medicine, University College London, London WC1E 6BT, UK; 4 Hunan Vocational Institute of Safety Technology, Changsha 410151, China; lha_sunny@hotmail.com; 5 Zhuzhou Teachers College, Zhuzhou 412000, China; 6 Testing Center of Hunan Academy of Forestry, Changsha 410004, China

**Keywords:** camellia seed oil, volatile flavor compounds, headspace solid-phase microextraction, gas chromatography–mass spectrometry, principal component analysis

## Abstract

In this study, the flavor compounds of Camellia seed oils obtained by four processes were characterized by headspace solid phase microextraction/gas chromatography/mass spectrometry (HS-SPME/GC/MS). A variety of about 76 volatile flavor compounds were identified from all the oil samples. Of the four processing processes, the pressing process can retain a lot of volatile components. Among these, compounds nonanal and 2-undecenal were predominantly in the majority of the samples. Meanwhile, other compounds such as octyl ester formic acid, octanal and 2-nonenal (E), 3-acetyldihydro 2(3H)-furanone, (E)-2-decenal, dihydro-5-penty 2(3H)-furanone, nonanoic acid, and dodecane were also among the most consistently found compounds among the oil samples analyzed. The principal component analysis carried out to categorize the data produced seven clusters of the total oil samples based on the number of flavor compounds obtained in each sample. This categorization would lead to understanding the components which highly contributed to the characteristic volatile flavor and build up the flavor profile of Camellia seed oil.

## 1. Introduction

Camellia is one of the four main oil-bearing trees species in the world, along with olive, palm, and coconut. Depending on the environmental conditions, harvesting time, species, agronomic practices and cultivar [1], the seed oil content of traditional Camellia varieties can vary between 24% and 50%, with an average of 30% [2]. Oil is obtained from all Camellia species, whereas *Camellia oleifera* (*C. oleifera*) was applied for its edible oil. Oil-tea camellia (*C. oleifera* Abel) is a subtropical evergreen shrub or small tree and grows naturally from 18° to 34° N and in acidic soils with a pH of 4.5–6.0 [1]. It has been widely cultivated in more than 10 provinces in southern China. The annual production of Chinese camellia oil has exceeded 600,000 tons by the end of 2017, which is important for the economy of southern China [3]. Some other Asian countries (i.e., Vietnam) have also started cultivating *C. oleifera* widely [4]. 

Camellia seed oil from high-quality seeds is commonly used in China as an edible oil and traditional medicines [5]. Camellia seed oil is highly nutritious and chemically similar to olive oil, containing high amounts of oleic acid and linoleic acid, but it is low in saturated fats [6]. Camellia seed oil has a variety of biological properties including antioxidant, anticancer, blood cholesterol regulating, beneficial to the digestive system and immunomodulatory activities [7,8]. Camellia seed oil and its by-products are also used in the production of paint and fertilizer, soaps, hair oils, lipstick, anti-wrinkle creams, and sunscreens [5]. Several common methods of oil extraction from camellia seeds have been reported, including organic solvent extraction, steam explosion pretreatment extraction, cold-pressure extraction, subcritical water extraction, ultrasonic-assisted, microwave puffing pretreatment and water enzyme extraction [9,10,11,12,13]. Additionally, several recent studies have reported the physiochemical properties, fatty acid profile and tocopherol compositions of the oil from *C. oleifera* Abel cultivated in various provinces of China [14,15,16]. 

However, camellia saponin has been reported have foam-stabilizing and emulsifying properties [17,18], which might prevent the direct extraction of oil from *C. oleifera* seeds. Meanwhile, the rapid increase in demand for camellia seed oil in recent years has driven up price and contributed to the more widespread use of cheaper or lower-quality edible vegetable oil for adulteration or mislabeling [19,20,21]. The planting area and output of Camellia seed oil in China are still very limited. In addition, the processing link of camellia seed oil in China lacks scientific planning and backward management. There are no specific industry standards regarding the technical parameters for processing camellia seeds, and the processing conditions are very arbitrary and uncertain. This problem may affect the quality indicators and flavor quality of camellia seed oil, and it can even lead to unacceptable organoleptic flavors for consumers. Furthermore, the scarcity of information data on the characteristics of oil and tea seed variety resources poses a serious challenge in determining the authenticity of camellia seed oil.

Various analytical methods have been developed to determine the adulteration of various high-priced oils, including Fourier Transform Infrared Spectroscopy (FTIR), stable carbon isotope technology, near-infrared spectroscopy (NIR), headspace–mass spectrometry, mid-infrared spectra, low field nuclear magnetic resonance (LF-NMR), high-performance liquid chromatography (HPLC) and gas chromatography-mass spectrometry (GC–MS) [22]. The distinctive flavor of camellia seed oil is attributed to a large number of different classes of compounds, such as aldehydes, alcohols, esters, hydrocarbons, ketones, furans and other volatile compounds that have not yet been identified. A previous study has reported to detect the adulteration of camellia seed oil with soybean oil by gas chromatography–mass spectrometry (GC–MS) using parameters of total content of oleic and linoleic acid, the oleic to linoleic acid ratio and the content of linolenic acid [22]. However, only a few studies have reported the application of GC-MS combined with headspace solid-phase microextraction (HS-SPME) to characterize the complex volatiles of camellia seed oil and to detect the adulteration of camellia seed oil.

Therefore, the aim of this study was to use HS-SPME-GC-MS to separate and identify the characteristic volatile profile, evaluate and control quality in the production and processing process of pure Camellia seed oil.

## 2. Results and Discussion

### 2.1. The Composition of Flavoring Substances of Camellia oleifera

About 43 Camellia seed oil samples were collected from the commercial markets of Hunan province, China. The present investigation was aimed to address the lack of data about volatile flavor compounds/components of Camellia seed oil, which is the oil consumed by major population of China. The oil samples were extracted and analyzed using HS-SPME-GC/MS to determine the flavor profile. This analysis not only provided the flavor profile of the oil but also contributed to understand the quality of oil during processing and adulteration, if any. Furthermore, the flavor compounds were subjected to PCA and HCA to categorize the majorly seen compounds among the obtained 76 compounds. 

**Figure 1:**
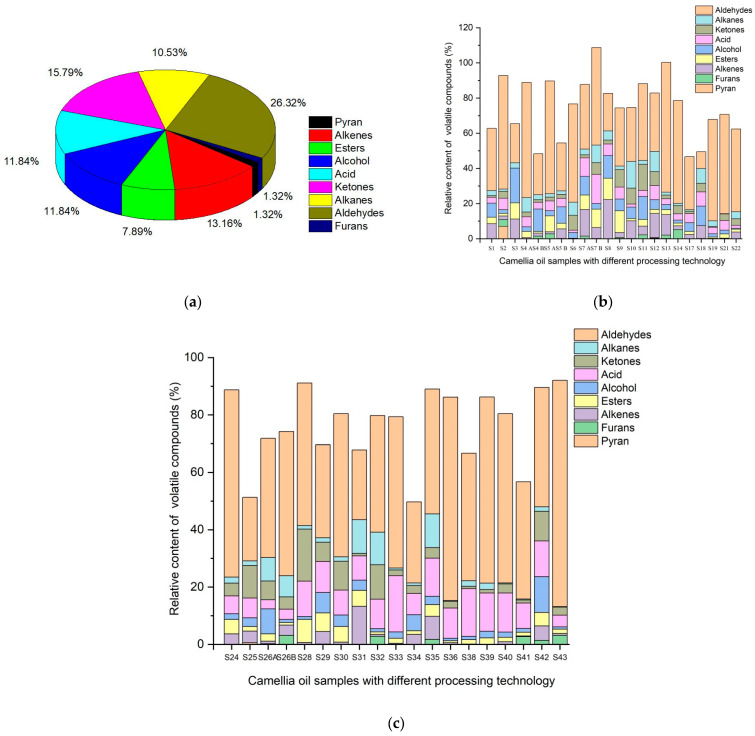
The composition and content of volatile compounds in all Camellia seed oil (**a**) and single sample (**b**,**c**).

The GC-MS method was used to separate and identify 76 volatile compounds (Figure 1) with SI (similarity) > 80 from camellia oil prepared by the four methods, including 1 pyranyl, 9 alcohols, 1 furan, 20 aldehydes, 9 acids, 12 ketones, 8 alkanes, 10 alkenes, and 6 esters. Their relative compositions are shown in Figure 1, among which aldehydes account for the largest proportion, up to 26.32%, which is followed by ketones 15.79%, alcohol 11.84%, acid 11.84%, alkenes 13.16%, alkanes 10.53%, esters 7.89%, pryan 1.32%, and furans 1.32%.

### 2.2. Characterization of Volatile Flavor Compounds Analyzed by HS-SPME–GC/MS

It can be seen from Table 1 that different processes have a certain impact on the categories and the number of volatile substances in the camellia seed oil. Among them, there were 71 volatile components belonging to 9 categories in PFO, 52 volatile components belonging to 8 categories in AEO, 44 volatile components belonging to 8 categories in SEO, and 76 volatile components belonging to 9 categories in RFO. Pyran was not detected by either the AEO or SEO. Most of the compounds identified in our study were reported in earlier investigations of Camellia seed oil samples in Yunnan and Zhejiang province, China [23].

Moreover, the processing technology also had a significant effect on the proportion of specific flavor substances. The contents of aldehydes, acids and alcohols were the highest and accounted for the largest proportion (23.33–55.14%, 3.58–16.61%, and 1.08–12.72%, respectively) in PRO. The main volatile components in the AEO were aldehydes, ketones and alcohols, accounting for 43.65–47.17%, 3.7–14.9% and 0.59–13.08%, respectively. In the SEO, aldehydes accounted for the largest proportion, reaching 78.87%, while ketones and acids were much less than those in other processes, only accounting for 2.79% and 4.06%, respectively. In the RFO, aldehydes, alcohols and acids accounted for 9.47–58.96%, 0–19.81% and 1.28–19.67%, respectively.

The processing technology also has a great influence on the composition proportion of each class of compounds. The compounds that are identified in most samples are discussed in Table 1. It can also be seen from Table 1 that nonaldehyde, octanoic aldehyde and hexanal are also present in the camellia seed oil of the four processing processes, indicating that nonaldehyde, octanoic aldehyde and hexanal are the main flavor substances affecting the camellia oil. This is consistent with previous reports [24] that aldehyde is the most important flavor substance, and aldehyde also produces a characteristic odor to oxidized fat and food, but a alow content of aldehyde may produce a characteristic aroma. Most aldehydes have a low threshold and can have a positive effect on oil flavor [25]. In particular, unsaturated aldehydes are very important flavor substances. Saturated aldehydes with low carbon number usually produce unpleasant pungent odor in accordance with them. On the whole, the content of flavor substances in the press was the most abundant, while the solvent extraction was the least, but the solvent extraction of aldehydes was the most, accounting for 78.87%, which may be due to the reduction in other substances, while the relative proportion of aldehydes was increased.

Nonanal was the most predominantly found compound and showed significantly high levels in most of the identified samples. Its presence was noted in about 41 samples and their peak area percentage in fresh pressing, hydro-enzymatic, refining, and solvent extraction methods was 6.68–14.67, 9.29–10.37, 4.99–19.78, and 10.66, respectively. This was in agreement to previous work by Kim et al. [26], who identified 11 volatile compounds in the camellia seed oil, and nonanal was one of them. 

Hexanal accounted for the second highest proportion of aldehydes in the PRO, and their peak area percentage was 4.25–10.08. Hexanal also had a higher proportion in the AEO, RFO and SEO, which were 4.66–21.47, 1.577–19.53 and 7.74, respectively. Octanal is the third highest proportion of aldehyde compounds, their proportion in the AEO, RFO and SEO was 2.54–9.93, 4.85–10.42%, 2.09–17.17%, which was lower than that of SEO (8.14%), and the proportion of 2-decenal, (E)-, 2-undecenal and 2, 4-decadienal in solvent-extracted camellia oil was also significantly lower than those obtained by the other three methods.

However, the number of alcohol compounds in the SEO was significantly less than that of the others. A total of nine alcohol compounds were detected in all the oils, among which nine alcohol compounds were detected in PRO and RFO, while there were more than the eight alcohol compounds in AEO and five alcohol compounds in SEO. Most alcohols have the fragrance of flowers and plants. Although the threshold of alcohols is high, alcohols can assist other flavor components to make the overall flavor of the sample richer and stronger. Similarly, the quantity and content of ketone compounds are also significantly different in different processing oil. A total of 12 ketones were detected in all oils, there were 10, 8, 10 and 6 ketones in PRO, AEO, RFO and SEO, respectively. Ketones are derived from the Maillard reaction or the further oxidation of aldehydes. Although their threshold value is high, they can still affect the flavor of oil, mainly because ketones can assist other flavor components to make the overall flavor richer and stronger, and most ketones are fragrances and creaminess. Similarly, six esters were detected in the PRO and RFO, which was significantly higher than five esters in the EAO and three esters in the SEO. Ester compounds generally have wine, floral and a typical fruit aroma, and they are a very important kind of fragrance substance. They are usually generated by lipid metabolism or the esterification reaction of acids and alcohols, and the threshold of esters was low and had a great impact on the overall flavor of vegetable oil [27]. The same trend was observed in other alkenes and alkanes compounds. To sum up, it is generally believed that the flavor of PRO is better than that of EAO, SEO and RFO, which may be related to the comprehensive effect of volatile flavor formation, as the type, content, and sensory threshold of these components and their accumulation, separation, inhibition, synergy and other effects objectively affect the content and quality of aroma in edible oil.

### 2.3. Principal Component Analysis (PCA), Hierarchical Cluster Analysis (HCA) of GC–MS Data

Overall, 76 volatile flavor components were identified from all the oil samples analyzed in the study. Therefore, to categorize all the flavor compounds and their presence in the samples, PCA was conducted. By projecting the objects of the dataset into the space of the first few components, it is possible to visualize the differences among the various objects. PCA can summarize the data based on their similarities and differences by reducing the number of dimensions without much loss of information. Therefore, in the present study, PCA was applied to determine which were the most samples based on the presence of flavor compounds.

**Figure 2:**
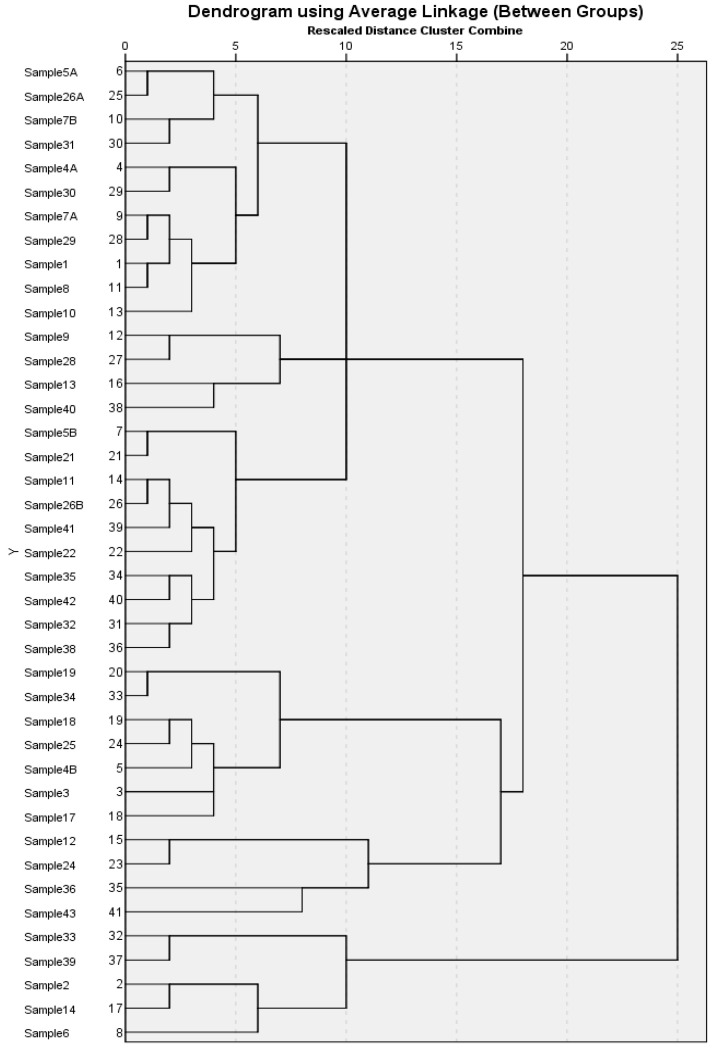
Camellia seed oil cluster map based on volatile components.

In our analysis, we consider the principal components based on the eigenvalues which are shown in Table 2. Considering this rule, we extracted seven principal components. The principal components PC1 and PC2 produced 42% of the variance explanation, while PC3 produced 4.29% (Table 2). The PCA with seven factors (nearly 67% of the total variance) discriminates between the 41 samples, which leads us to conclude that it is possible to successfully differentiate samples from different samples according to their compounds. According to the ‘scree plot’ presented in Figure 2, a total of seven clusters were formed. The 1st cluster included samples 5A, 7B, 26A, and 31, while samples 1, 4A, 7A, 8, 10, 29, and 30 were included in the 2nd cluster. The samples 9, 13, 28, and 40 were included in the 3rd cluster, and the 4th cluster included samples 5B, 11, 21, 22, 26B, 32, 35, 38, 41 and 42. The 5th and 6th cluster included the set of samples 3, 4B, 17, 18, 19, 25, 34, and 12, 24, 36, and 43, respectively, while the last 7th cluster included samples 2, 6, 14, 33, and 39. 

Like PCA, cluster analysis (CA) is another method of determining differences among various samples by dividing all samples into groups (clusters) according to similarity and finding the similarity among objects in a multidimensional space, forming clusters between the nearest objects. Ward’s method as the amalgamation rule and the squared Euclidean distance as a metric were used to establish clusters. Here, we focus on hierarchical clustering, one of the most widely used clustering algorithms, which categorizes observations into a hierarchical set of groups organized in a tree-like structure called a dendrogram. Hierarchical clustering produces a dendrogram that represents a nested set of clusters. The dendrogram of the hierarchical cluster analysis (HCA) results is shown in Figure 2; all samples were divided into seven groups, which is basically the same with the results of PCA. 

On the whole, it can be depicted that the oil samples in the 1st cluster (5A, 7B, 26A, and 31) contained the highest number of the volatile flavor compounds, and their profile can be considered the ideal profile for Camellia seed oil. This profile can be used to compare and understand the quality of any unknown oil sample after the analysis of their flavor compounds. The last cluster, the 7th cluster, contained samples (2, 6, 14, 33, and 39) with lesser flavor compounds in them, indicating the low quality of the oil. This may occur due to losses during postharvest processing and storage (temperature, moisture content and humidity) of oil seeds, which affect the quality of the bioactive compounds in the extracted oil [28]. 

## 3. Materials and Methods

### 3.1. The Preparation of Oil with Different Processing Methods

A total of 43 *C. oleifera* oil samples were provided by the Testing Center of Hunan Academy of Forestry (Changsha, China), which have been processed from the *C. oleifera* seeds in several steps. All *C. oleifera* seeds were initially crushed with a pulverizer; then, they were sorted with a standard sieve, achieving the size of the seeds ranging from 296 to 1200 mm. The *C. oleifera* seeds were finally dried in a drying oven (101-2AB, Tianjin Taiste Instrument Co., LTD, Tianjin, China) at 60 °C for 4–6 h, cooled to a constant weight, then sealed and stored. After that, 5 oil samples were produced by pressing, 32 were produced by refining and 2 were produced by the aqueous-enzymatic method. The oil sample extracted from the pressing (PRO) has several steps as follows: The shells and kernels of the pre-treated seeds were crushed separately by pulverizer (WJX-200,Shanghai Yuanwo Industry and Trade Co. LTD, Shanghai, China) and sorted through a 2 mm sieve; then, they were mixed proportionally into embryos, steamed at high temperature (80 °C) and pushed into a physical press to extract the oil. The refined oils (RFO) were obtained by a standardized oil refining process based on the physical pressing. The oil samples produced from solvent extraction (SEE) include several steps as follows: 50 g of pretreated seeds powder was extracted by 400 mL of petroleum ether (Sinopharm Chemical Reagent Co., Ltd. Shanghai, China) as solvent for 60 min. The extracted oil was refined using a rotary evaporator and dried in vacuum to a constant weight. For the aqueous enzymatic oils (AEO), the pre-treated seeds were first mechanically crushed and ground and then hydrolyzed with cellulase, pectinase, protease and others. Finally, the oil samples were prepared by de-sludging, oil–water separation and filtration. 

### 3.2. Chemicals and Reagents of Flavor Collections

The manual sampling of oil was carried out by SPME Fiber holders (Supelco, Bellefonte, PA, USA). SPME Fiber Carboxen/DVB/PDMS 50/30 2 cm (Supelco, Bellefonte, PA, USA) was performed to adsorb the flavors of oil samples. The GC-MS method (GCMS-QP2010 Ultra (Shimadzu, Kyoto, Japan)) was performed to inject the flavor.

### 3.3. HS-SPME Analysis

First, 2 mL of each oil sample was weighed and filled into the headspace flask and then was sealed with a spacer. The headspace flask containing the samples was heated in a water bath while maintaining a constant temperature of 40 °C for 20 min. The activated SPME extraction head (270 °C, 1 h) was subsequently inserted through the spacer. The headspace was absorbed at 70 °C for 40 min. Finally, the GC injector was inserted and analyzed at 250 °C for 3 min.

### 3.4. GC–MS Analysis

The GC-MS analysis was performed according to the process described by Wang et al. [29]. Gas chromatography conditions: DB-5MS, elastic capillary column (30 m × 0.25 mm × 0.25 m, Agilent, Santa Clara, CA, USA), temperature rising procedure: 40 °C, 2 min, increased to 220 °C at 5 °C/min, and held for 10 min, Inlet temperature: (maintain) 250 °C. The high-purity helium was employed as the carrier gas at a flow rate of 1 mL/min with a no-shunting (no dilution) injection mode. Mass spectrometry conditions: electron bombardment ion source (EI), electron energy 70 ev, filament emission current 200 A; ion source temperature 150 °C; interface temperature: 280 °C; full scan mode, scanning quality range *m*/*z* 35–350 amu (molecular mass) sieving.

### 3.5. Qualitative and Quantitative Detection of Volatile Compounds

The GC/MS software with an NIST 17 standard library was used to automatically retrieve the mass spectrometric data of each component, and the chemical composition was determined according to the mass spectrometric splitting law. The qualitative analysis was carried out for the substances whose matching degree was not less than 80%, and then the relative content of each component was calculated by the peak area normalization method for quantitative analysis.

### 3.6. Statistical Analysis

The data obtained from HS-SPME–GC/MS analysis were recorded to document the various volatile flavor components obtained in individual samples. The data were separated to record the peak area of each flavor component in each sample. Additionally, statistical analysis on flavor compounds was performed by using SPSS 21(SPSS Inc., Chicago, IL, USA). For different samples, the variables were the identified volatile compounds, and the input values in the matrix were the relative abundance of the volatile compounds. The raw input data were used for the principal component analysis (PCA). Hierarchical cluster analysis (HCA) was also used to identify the different clusters based on the similarity of compounds, looking for similarities between objects in a multidimensional space to form clusters between the nearest objects. The method of Ward was used as a merging rule, and the square of the Euclidean distance was used as a metric to create clusters. PCA and HCA were comprehensive evaluation methods which were used to clustering the data as per the flavors. The method started with a PCA of the samples, and then, we extracted several principal components as variables for the clustering analysis. For the factor analysis and HCA, 41 samples were included, while 2 samples were excluded, namely samples 16 and 27, since most of the compounds in these two samples were missing.

## 4. Conclusions

The processing technology has a significant impact on the volatile components in camellia oil, which is mainly reflected in the number of volatile components and the difference of specific constituent types as well as the proportion of each volatile compound. The results of this study showed that the pressing oil and the refined oil based on pressing oil as raw materials can maintain a wide variety of volatile components, especially alcohols, ketones and alkaloids, which are much higher than those of the aqueous enzymatic oil and solvent extracted oil. In addition, the results of this study showed that the aroma of camellia seed oil is not manifested by one or a few compounds but by the synergistic effect of various components, reflecting different characteristic aromas. In production, this method can be used to analyze the composition and content of flavor substances of camellia seed oil, and it can also be used to evaluate the camellia grade combined with sensory analysis so as to improve the production technology. In subsequent studies, more sample data can be collected, and we can combine the metabolomics approach to reveal the generation rule and mechanism of volatile flavor substances of camellia seed oil and establish screening technology based on volatile flavor substances to provide a scientific basis for the quality control and adulteration evaluation of camellia seed oil.

## Figures and Tables

**Table 1:** Volatile flavor compounds and their peak area percentage in different processing technology of camellia oil (%).

Compounds	Chemical Functional Group Classification	PRO	AEO	RFO	SEO
Formic acid, octyl ester	Esters	0.91–1.91	1.62–2.06	0.17–4.3	0.9
Ethyl tiglate	Esters	0.63–2.26	0–0.63	0.03–3.4	0
Hexadecanoic acid, methyl ester	Esters	0–0.02	0–0.01	0.02–7	0.04
2-Hexenoic acid, ethyl ester	Esters	0.54–3.45	0–0.7	0.19–3.29	0
1-Octen-3-ol, methyl ether	Esters	0.34–2.27	0–0	0.11–7	0.6
Formic acid, heptyl ester	Esters	0.51–0.6	0–0.41	0.46–4.93	0
Esters subtotal		0.91–10.4	1.62–3.81	1.54	0–12.38
Styrene	Alkenes	0.81–5.89	0–4.72	0.54–14.9	0
3,5-Octadien-2-one	Alkenes	0.13–2.14	0–1.19	0.04–7	0.09
4,6-Decadiene	Alkenes	0.08–0.15	0.08–0.08	0.04–7	0.14
3-Octen-2-one	Alkenes	0.3–0.31	0	0.11–2	0.19
Bicyclo [3.1.1]hept-2-ene, 2,6-d	Alkenes	0.04–0.06	0–0.08	0.03–7	0
Z-(13,14-Epoxy)tetradec-11-en-1	Alkenes	0–0.09	0	0.04–0.16	0
1,2-Dimethyl-4-oxocyclohex-2-en	Alkenes	0–0	0–0.27	0.06–0.29	0.21
Naphthalene, 2-methyl-	Alkenes	0–0	0–2.58	0.05–0.91	0
1H-3a,7-Methanoazulene, 2,3,4,7	Alkenes	0.05–0.16	0–0	0.09–7	0
3-Heptadecene, (Z)-	Alkenes	0–0.15	0–0	0.04–0.18	0.11
Dodecane	Alkanes	0.15–0.37	0.57–2.56	0.05–7	0
Tetradecane	Alkanes	0.11–0.14	0–0.18	0.03–0.66	0
Hexadecane	Alkanes	0.06–7	0.06–1.34	0.04–7	0
Pentadecane	Alkanes	0–7	0–0	0.03–7	0
2-Bromo dodecane	Alkanes	0.06–0.18	0–0	0.08–7	0.15
Heptadecane	Alkanes	0–0.18	0–0.06	0.03–7	0.05
Ethyl 1-methylcyclopropanecarbo	Alkanes	1.17–2.78	0–1.28	0.17–4.25	0
Oxirane, octyl-	Alkanes	0.05–0.08	0–0	0.08–7	0
Alkanes subtotal		1.64–6.29	3.77–5.07	0.6	0–11.78
2(3H)-Furanone, dihydro-5-penty	Ketones	0.17–0.3	0–0.19	0.06–2.36	0.31
Cyclohexanone, 2-(1-methyl-2-ox	Ketones	0.09–0.36	0–0.13	0.06–7	0.33
2-Sec-Butylcyclohexanone	Ketones	0.12–0.14	0–7	0.05–0.71	0.23
2-Dodecanone	Ketones	0.46–5.22	0.33–0.74	0.24–4.58	0.73
2(3H)-Furanone, 3-acetyldihydro	Ketones	0.21–0.75	0–0.25	0.08–7	0.49
2-Nonanone	Ketones	0.53–1.63	0–2.59	0.33–7.42	0.7
2-Pentadecanone	Ketones	0–0	0–0	0.02–7	0
2-Decanone	Ketones	0.29–0.35	0–0	0.11–5.43	0
2H-Pyran-2-one, tetrahydro-6-pr	Ketones	0.09–0.1	0–7	0.1–2.62	0
Ethanone, 1-(1H-pyrrol-2-yl)-	Ketones	0.48–1.21	0–0	0.08–3.23	0
.gamma.-Chlorobutyrophenone	Ketones	0–0.18	0–0	0.05–0.59	0
2-Pentadecanone, 6,10,14-trimet	Ketones	0–0	0–0.38	0.03–7	0
Ketones subtotal		1.57–6.72	3.7–14.9	2.79	0.79–18.16
Nonanoic acid	Acid	0.25–4.45	0.63–1.63	0.25–11.13	2.19
Octanoic acid	Acid	1.05–2.68	0–0	0.58–7	1.19
2-Butenoic acid, 2-methyl-, 3-m	Acid	1.02–2.03	0–1.51	0.39–4.18	0.41
Cyclopentanecarboxylic acid, 2-	Acid	0.15–0.66	0–0.21	0.13–0.74	0
Heptanoic acid	Acid	0–1.12	0–1.07	0.12–1.51	0
Nonanoic acid, pentafluoropheny	Acid	0.06–0.19	0–0	0.06–0.36	0
3-Methyl-2-butenoic acid, hepta	Acid	0.79–4.42	0–0	0.23–4.18	0.23
Hexanoic acid	Acid	0–1.56	0–0	0.39–3.67	0
3-Cyclopentylpropionic acid, 2-	Acid	0–7	0–0	0.03–1.27	0.04
Acid subtotal		3.58–16.61	1.7–3.35	4.06	1.28–19.67
Nonanal	Aldehydes	6.68–14.67	9.29–10.37	4.99–19.78	10.66
Octanal	Aldehydes	2.54–9.93	4.85–10.42	2.09–17.17	8.14
2-Decenal, (E)-	Aldehydes	1.32–2.77	3.49–3.49	0.86–9.53	11.94
2-Heptenal, (Z)-	Aldehydes	0.96–1.59	0.96–4.02	0.46–7.99	1.21
2-Nonenal, (E)-	Aldehydes	0.56–3.08	0.88–1.3	0.49–10.09	1.99
Heptanal	Aldehydes	1.03–3.03	2.68–3.09	0.24–6.37	1.58
2-Octenal, (E)-	Aldehydes	0.89–2.58	1.21–1.26	0.36–4.83	2.32
Dodecanal	Aldehydes	0.43–1.14	0–0.51	0.23–7	1.01
Hexanal	Aldehydes	4.25–10.08	4.66–21.47	1.57–19.53	7.74
2-Undecenal	Aldehydes	0.51–2.21	1.59–1.74	0.48–7	7.44
4-Oxononanal	Aldehydes	0.12–7	0–0	0.04–7	0.45
2,4-Nonadienal, (E,E)-	Aldehydes	0.34–0.65	0–0.17	0.08–1.08	0.38
2,4-Decadienal	Aldehydes	0–1.51	0–0.7	0.32–3.53	14.4
2,4-Decadienal, (E,E)-	Aldehydes	0.46–4.65	0.47–0.72	0.26–14.11	6.81
Benzaldehyde	Aldehydes	0.68–2.88	0–4.02	0.5–7.74	1.34
Tetradecanal	Aldehydes	0.03–0.04	0–0	0.03–7	0
2,4-Heptadienal, (E,E)-	Aldehydes	0.71–8.18	0–0.95	0.21–5.62	1.23
trans-4,5-Epoxy-(E)-2-decenal	Aldehydes	0–0.18	0–0	0–0.3	0.18
2,4-Dodecadienal	Aldehydes	0–0	0–0	0.02–7	0.05
Benzeneacetaldehyde, .alpha.-et	Aldehydes	0.09–0.09	0–0	0.05–0.34	0
Aldehydes subtotal		23.33–55.14	43.65–47.17	78.87	9.47–58.96
Furan, 2-pentyl-	Furans	1.51–3.09	0–2.13	0.27–5.15	3.18
1-Heptanol	Alcohol	0.42–1.23	0–1.7	0.32–2.95	0.48
1-Octen-3-ol	Alcohol	0.33–0.45	0–0.75	0.13–3.12	0.2
Phenylethyl Alcohol	Alcohol	0.21–2.38	0–1.25	0.33–16.19	0
1-Nonanol	Alcohol	0.09–0.35	0–0	0.06–1.38	0.06
Benzyl alcohol	Alcohol	0.25–0.25	0–0.18	0.11–1.67	0
n-Nonadecanol-1	Alcohol	0.08–0.27	0–0.59	0.03–7	0.06
3-Phenylpropanol	Alcohol	0.14–0.16	0–7	0.04–7	0
Phenol, 2,6-dimethoxy-	Alcohol	1.51–2.33	0–0.13	0.12–7	0
Mequinol	Alcohol	4.79–9.07	0–2.07	0.29–4.75	0
Alcohol		1.08–12.72	0.59–13.08	0.8	0–19.81
1H-2-Benzopyran-1-one, 3,4-dihy	Pyran	0.1–0.11	0–0	0.04–7	0

**Table 2:** Results of the principal component analysis.

Principal Components	Eigen Value	Total % of Variance	Cumulative % of Variance
1	17.318	42.238	42.238
2	3.368	8.215	50.453
3	1.761	4.295	54.748
4	1.434	3.498	58.245
5	1.309	3.192	61.437
6	1.151	2.807	64.244
7	1.109	2.705	66.950

## Data Availability

The data are available upon reasonable request.

## Sample Availability

Samples of the compounds are not available from the authors.
